# Histopathological Subtypes of Cutaneous Melanoma: Prognostic and Molecular Implications

**DOI:** 10.7759/cureus.90670

**Published:** 2025-08-21

**Authors:** Hussein Qasim, Mohammad Abu Shugaer, Karis Khattab, Matteo Luigi Giuseppe Leoni, Giustino Varrassi

**Affiliations:** 1 Department of Pathology and Laboratory Medicine, Jordan University of Science and Technology, Irbid, JOR; 2 Department of Pathology, Jordan University of Science and Technology, Irbid, JOR; 3 Department of Medicine, Yarmouk University, Irbid, JOR; 4 Department of Medical and Surgical Sciences and Translational Medicine, Sapienza University, Rome, ITA; 5 Department of Pain Medicine, Fondazione Paolo Procacci, Rome, ITA

**Keywords:** braf, cutaneous melanoma, histopathological subtypes, molecular implications, nf1, nras

## Abstract

Cutaneous melanoma is a biologically diverse and clinically aggressive malignancy with distinct histopathological subtypes that significantly influence its diagnosis, prognosis, and management. This comprehensive review explores the major melanoma subtypes, superficial spreading, nodular, lentigo maligna, acral lentiginous, and desmoplastic melanoma, along with rarer variants such as spitzoid, nevoid, and mucosal melanomas. Each subtype exhibits unique morphological characteristics, growth patterns, anatomical distribution, and molecular profiles, including variations in key mutations such as B-Raf proto-oncogene, serine/threonine kinase (BRAF), KIT proto-oncogene receptor tyrosine kinase (KIT), Neuroblastoma RAS viral oncogene homolog (NRAS), and Neurofibromin 1 (NF1). While histologic subtype is not incorporated into formal staging systems, it frequently correlates with tumor behavior and patient outcomes, guiding surgical planning and adjuvant therapy decisions. Advances in immunohistochemistry, molecular diagnostics, and genomic profiling have refined melanoma classification and opened new avenues for targeted and immune-based therapies. However, diagnostic challenges persist due to overlapping features and under-characterization of rare variants. This review underscores the need for a multimodal approach that integrates histopathologic, molecular, and clinical data to achieve precise classification and optimize patient care in the era of personalized oncology.

## Introduction and background

Cutaneous melanoma (CM) is a malignant tumor of melanocytes and remains one of the most aggressive forms of skin cancer [1]. Although CM accounts for only about 1.7% of all skin cancer cases, it is responsible for the majority of skin cancer-related deaths due to its high metastatic potential [2]. The global incidence of CM has increased substantially over the past three decades. The age-standardized incidence rate rose from approximately 3.0 cases per 100,000 in 1990 to 3.56 per 100,000 in 2021, representing a 19.3% increase, while the number of new cases increased from roughly 124,300 to 303,100 during the same period, a 143.8% rise globally [3]. Ultraviolet (UV) radiation remains the most significant environmental risk factor [3]. Early-stage melanomas are highly curable with surgical excision, but advanced-stage disease presents a significant therapeutic challenge [4].

Histopathological classification plays a pivotal role in melanoma diagnosis, prognosis, and treatment planning [5]. The major histological subtypes, superficial spreading melanoma (SSM), nodular melanoma (NM), lentigo maligna melanoma (LMM), acral lentiginous melanoma (ALM), and desmoplastic melanoma (DM), differ not only in morphology and anatomical distribution but also in patient demographics, tumor behavior, and genetic alterations [6]. These subtypes were first introduced in the mid-20th century, and while their definitions have evolved, they remain essential in contemporary clinical practice [7]. In recent years, the integration of molecular and genetic insights has refined our understanding of these subtypes [8]. The WHO’s latest melanoma classification emphasizes the importance of solar elastosis, UV-induced mutations, and anatomical site, and categorizes melanomas based on cumulative sun damage (CSD) and genetic profiles [9]. For instance, the B-Raf proto-oncogene, serine/threonine kinase (BRAF) mutations are common in melanomas arising on intermittently sun-exposed skin (e.g., SSM), whereas KIT proto-oncogene receptor tyrosine kinase (KIT) and Neurofibromin 1 (NF1) mutations are more prevalent in acral and desmoplastic melanomas, respectively [10]. These distinctions not only aid diagnosis but also help tailor systemic therapies such as targeted agents and immunotherapy [11]. Despite histological subtype not being part of formal staging systems like American Joint Committee on Cancer (AJCC), it often correlates with prognosis; SSM and LMM generally portend better outcomes, while NM and ALM are associated with higher mortality, partly due to delayed diagnosis and biological aggressiveness [12,13]. Moreover, subtypes like DM, with unique features such as neurotropism and low nodal metastatic risk, require individualized surgical and adjuvant strategies [13].

This review aims to provide a comprehensive, integrated understanding of CM subtypes by examining their histological characteristics, prognostic implications, molecular landscapes, and roles in therapeutic decision-making. Bridging histopathology with genomic and immunologic insights is essential for advancing personalized melanoma care.

## Review

Methodology

A comprehensive literature search was conducted using PubMed, Scopus, and Google Scholar to identify relevant publications on cutaneous melanoma and its histopathological subtypes. Keywords included “cutaneous melanoma,” “histopathological subtype,” “superficial spreading melanoma,” “nodular melanoma,” “lentigo maligna melanoma,” “acral lentiginous melanoma,” “desmoplastic melanoma,” “rare melanoma variants,” “molecular profile,” and “prognostic implications.” Searches were limited to articles published in English, with a focus on the last 10 years to capture recent advances, though seminal older studies were included where foundational to the topic. Reference lists of key articles and relevant guidelines from the World Health Organization (WHO) and the American Joint Committee on Cancer (AJCC) were also reviewed to ensure completeness.

Histopathological subtypes of CM

**Figure 1:**
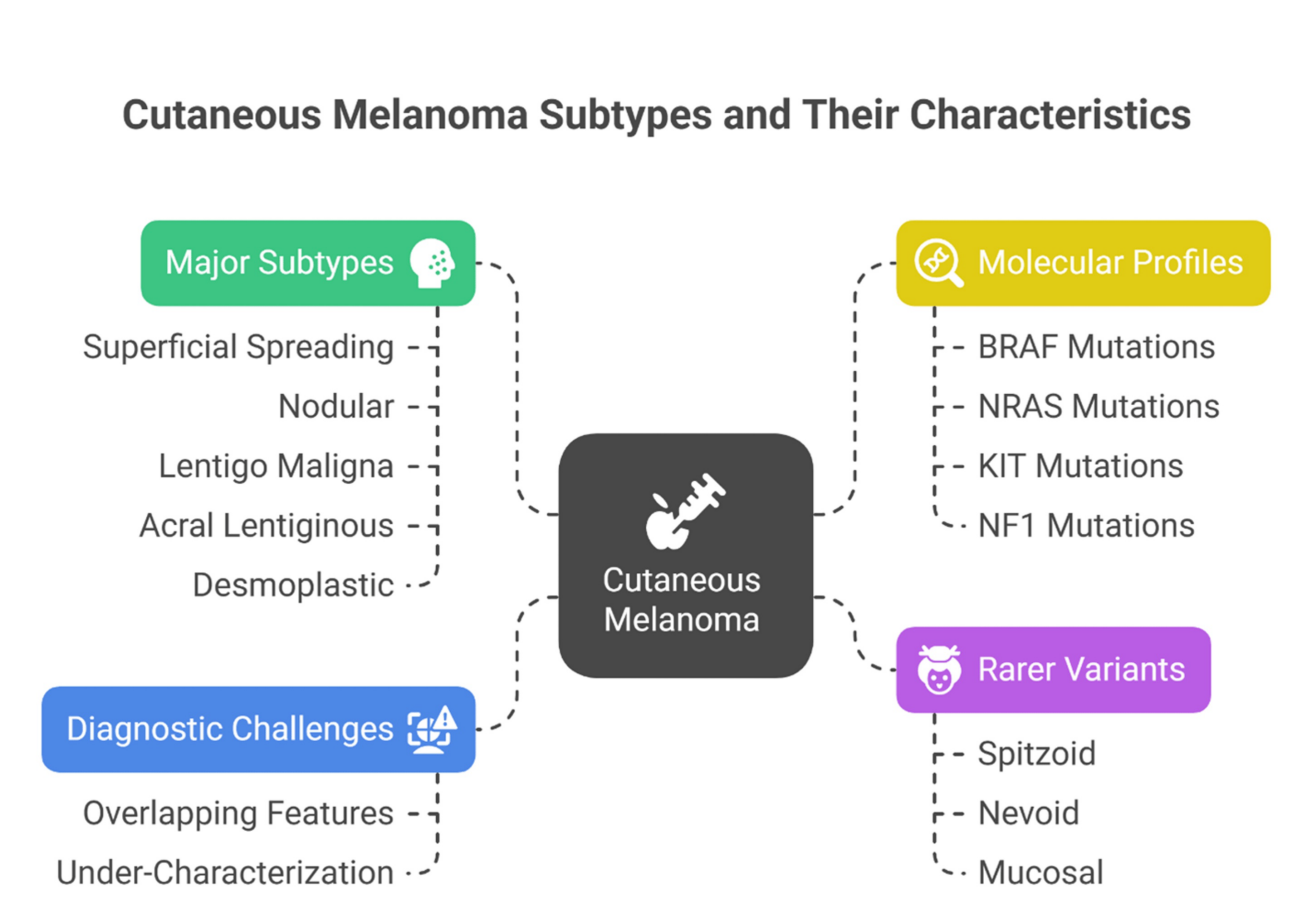
Overview of cutaneous melanoma subtypes and their associated characteristics, including major histological categories, molecular mutation profiles, rarer variants, and diagnostic challenges. BRAF: B-Raf proto-oncogene, serine/threonine kinase; KIT: KIT proto-oncogene receptor tyrosine kinase; NRAS: Neuroblastoma RAS viral oncogene homolog; NF1: Neurofibromin 1. Classification based on the WHO Classification of Skin Tumours, 4th Edition [9]. Image created by Dr. Karis Khattab on Microsoft Powerpoint (Microsoft Corp., Redmond, WA, US)

CM is classified into several distinct histopathological subtypes, each reflecting differences in clinical presentation, growth patterns, sun exposure, and molecular alterations [14]. Understanding these subtypes is essential for accurate diagnosis, prognostic estimation, and therapeutic planning (Figure 1).

**Figure 2:**
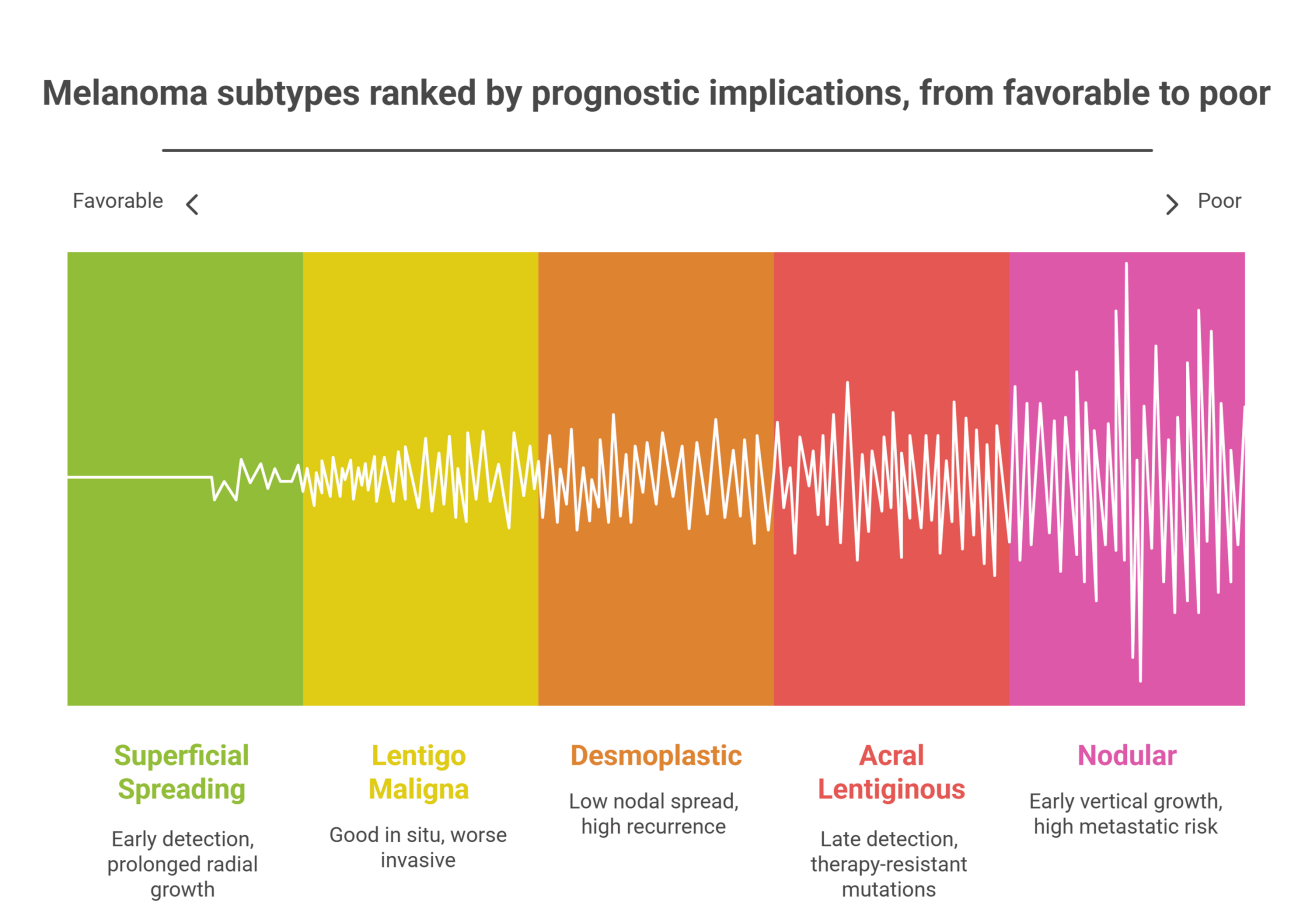
Melanoma subtypes ranked by prognostic implications, from favorable to poor Image created by Dr. Karis Khattab on Microsoft Powerpoint (Microsoft Corp., Redmond, WA, US)

Figure 2 illustrates the prognostic implications of the CM subtypes.

Superficial Spreading Melanoma (SSM)

SSM is the most prevalent CM subtype, accounting for approximately 60-70% of cases [15]. It commonly affects younger to middle-aged adults and typically arises on intermittently sun-exposed areas such as the trunk and proximal extremities [16]. Clinically, it presents as a slowly enlarging, asymmetrical pigmented lesion with irregular borders [17]. Histologically, it features a radial growth phase with pagetoid spread of atypical melanocytes in the epidermis, followed by eventual vertical invasion [17]. Molecularly, SSM is strongly associated with BRAF V600E mutations, found in nearly 50% of cases, and less commonly with Neuroblastoma RAS viral oncogene homolog (NRAS) mutations [18].

Diagnostically, SSM may be challenging in its early radial growth phase, as it can mimic benign pigmented lesions such as dysplastic nevi, particularly in cases with subtle asymmetry or partial regression [15]. Prognostically, SSM generally carries a favorable outcome when detected early due to its prolonged radial growth phase, allowing for curative excision; however, prognosis worsens significantly once vertical growth and dermal invasion occur [15].

Nodular Melanoma (NM)

NM represents 15-30% of melanomas and is characterized by an early and often exclusive vertical growth phase, leading to deeper invasion and worse prognosis [19]. It frequently appears as a uniformly pigmented or amelanotic nodule that enlarges rapidly and may ulcerate [20].

Histologically, NM lacks the radial growth phase seen in SSM and often demonstrates a high mitotic rate and ulceration [21]. BRAF and NRAS mutations are also common in NM, though with slightly different frequencies than SSM [22]. Due to its aggressive nature and often delayed detection, NM is associated with higher mortality.

Lentigo Maligna Melanoma (LMM)

LMM typically arises in older individuals on chronically sun-exposed areas such as the face, neck, and forearms [23]. It evolves from lentigo maligna, a melanoma in situ, over many years [24]. Clinically, it presents as a flat, slowly enlarging pigmented macule [24]. Histopathologically, LMM shows atypical melanocytes along the basal epidermis with epidermal atrophy and solar elastosis [25]. The transition to invasive melanoma occurs gradually [25]. Mutationally, it is less often associated with BRAF mutations and more frequently with NRAS and KIT alterations [26]. Diagnostically, early LMM can be challenging to distinguish from benign pigmented lesions such as solar lentigo or pigmented actinic keratosis, particularly in sun-damaged skin with irregular pigmentation. In addition, biopsy sampling errors may occur if the most atypical areas are missed, potentially delaying diagnosis [27]. Despite slow progression, once invasive, LMM may exhibit aggressive behavior if not treated early [27].

Acral Lentiginous Melanoma (ALM)

ALM accounts for 2-8% of melanomas and is the most common subtype in darker-skinned populations [28]. It occurs on acral sites, palms, soles, and nail beds, where UV exposure is minimal [29]. Clinically, ALM is often diagnosed late due to its hidden location and non-specific appearance [30]. Histologically, it features lentiginous growth with atypical melanocytes at the dermoepidermal junction and irregular invasion into the dermis [31]. KIT mutations and chromosomal instability are more commonly observed in ALM [32]. The prognosis tends to be poorer due to late detection and limited response to conventional targeted therapies [32].

Desmoplastic Melanoma (DM)

DM is a rare but distinct subtype, accounting for approximately 1-4% of all CMs [33]. It may appear as a firm, scar-like or amelanotic lesion and is frequently misdiagnosed [13]. Histologically, DM is marked by spindle-shaped melanocytes dispersed in a fibrous or desmoplastic stroma, often accompanied by neurotropism [34]. Immunohistochemical (IHC) markers like S100 and SOX10 are positive, while HMB-45 and Melan-A are frequently negative [35]. Genetically, DM is associated with high mutational burden and NF1 mutations and lacks common driver mutations like BRAF [36]. Although lymph node metastases are rare, local recurrence is more frequent, necessitating wide excision and consideration of adjuvant radiotherapy [37].

Other Rare Subtypes

Several other melanoma subtypes, though less commonly encountered in clinical practice, present distinct histopathological and clinical features [14]. Spitzoid melanoma resembles a Spitz nevus but displays cytological atypia and invasive behavior, often requiring molecular confirmation for diagnosis [38]. Nevoid melanoma mimics a benign nevus under the microscope, making it a diagnostic challenge due to its subtle yet malignant potential [39]. Mucosal melanoma, while not a cutaneous form, arises in mucous membranes and is considered in the differential diagnosis due to its aggressive nature and unique management needs [40]. Animal-type melanoma, also known as pigmented epithelioid melanocytoma, is heavily pigmented with a generally indolent course but uncertain malignant potential [41]. Lastly, primary dermal melanoma is confined to the dermis or subcutis without epidermal involvement, and although rare, it may carry a more favorable prognosis than conventional nodular melanoma [42]. Table 1summarizes the key clinical and histopathological distinctions among the major melanoma subtypes.

**Table 1:** Clinical and histopathological features of the major subtypes of cutaneous melanoma

Subtype	Common sites	Clinical features	Growth pattern	Histological features
Superficial spreading melanoma (SSM) [43]	Trunk, proximal extremities	Asymmetrical pigmented lesion	Radial → vertical	Pagetoid spread, atypical melanocytes in the epidermis
Nodular melanoma (NM) [44]	Trunk, head, and neck	Rapidly growing nodule, often ulcerated	Vertical (early onset)	No radial phase, high mitotic rate, ulceration
Lentigo maligna melanoma (LMM) [45]	Face, neck, forearms (elderly)	Slowly enlarging macule	Long in situ phase → vertical	Basal atypical melanocytes, solar elastosis
Acral lentiginous melanoma (ALM) [46]	Palms, soles, nail beds	Pigmented macule/patch, often detected late	Lentiginous, vertical	Junctional melanocyte proliferation, dermal invasion
Desmoplastic melanoma (DM) [13]	Head and neck	Firm, amelanotic scar-like lesion	Infiltrative fibrous pattern	Spindle cells in desmoplastic stroma, neurotropism

Prognostic implications of the histopathological subtypes

While traditional melanoma staging systems such as the AJCC primarily rely on tumor thickness (Breslow depth), ulceration, and mitotic rate, the histopathological subtype also provides significant prognostic information by influencing both tumor biology and the likelihood of early detection [47]. Table 2 provides a comparative summary of prognostic and treatment-related features of the major melanoma subtypes.

**Table 2:** Prognostic and therapeutic considerations for cutaneous melanoma, classified by subtype SSM: Superficial spreading melanoma; NM: Nodular melanoma; LMM: Lentigo maligna melanoma; ALM: Acral lentiginous melanoma, DM: Desmoplastic melanoma; BRAF: B-Raf proto-oncogene, serine/threonine kinase; KIT: KIT proto-oncogene receptor tyrosine kinase. Table based on the findings of the European consensus-based interdisciplinary guideline for melanoma [48].

Subtype	Prognosis	Common management considerations	Sentinel lymph node risk	Adjuvant therapy sensitivity
SSM	Favorable (if early)	Early detection, surgical excision	Moderate	BRAF-targeted, immunotherapy
NM	Poor	Rapid growth, early metastasis, wider margins	High	Often requires systemic therapy
LMM	Favorable (if in situ)	Early facial recognition, cosmetic surgical planning	Moderate	Immunotherapy if invasive
ALM	Poor	Often advanced at diagnosis, nail/plantar surgery complex	High	KIT inhibitors (if mutated)
DM	Variable (pure > mixed)	Low nodal spread, high recurrence, consider radiotherapy	Low	High response to immunotherapy

SSM generally carries a favorable prognosis when diagnosed early, owing to its prolonged radial growth phase [49]. These tumors are often thin (<1 mm) and detected during routine skin exams, allowing for curative surgical excision [50]. However, once the vertical growth phase initiates, the risk of metastasis increases notably [51]. In contrast, NM has one of the poorest prognoses among CMs [52]. It lacks a radial growth phase, leading to early dermal invasion and greater Breslow thickness at presentation [53]. This subtype frequently exhibits ulceration and a high mitotic index, both of which contribute to its aggressive metastatic potential [51]. LMM typically develops slowly and is often identified in situ as lentigo maligna, leading to relatively favorable outcomes [23]. However, if progression to invasive disease occurs, especially when overlooked in older patients or in cosmetically sensitive facial areas, it can result in significant morbidity and biological aggressiveness [54]. ALM is usually diagnosed at a more advanced stage due to its development in less visible regions such as the soles, palms, or beneath the nails [55]. As a result, ALM tends to have a poorer prognosis, compounded by diagnostic delays and a higher prevalence of chromosomal instability and therapy-resistant mutations [56]. Finally, DM presents a unique paradox: while it often demonstrates deep local invasion and neurotropism, it has a low tendency for regional lymph node metastasis [57]. Nonetheless, DM has a high risk of local recurrence and typically necessitates wide surgical margins [58]. Prognoses within DM subtypes vary; “pure” DM is associated with better outcomes than “mixed” forms, which may exhibit more aggressive behavior [13].

Prognostic factors across subtypes

Several histological and clinical features significantly influence prognosis across all cutaneous melanoma subtypes [59]. Among these, Breslow thickness remains the single most powerful and consistently validated predictor of patient survival [60]. Features such as ulceration, high mitotic rate, and lymphovascular invasion are indicative of more aggressive tumor behavior and are associated with worse outcomes [61]. Regression, which was previously thought to be a favorable finding due to the apparent immune-mediated destruction of tumor cells, now carries an uncertain prognostic significance, with some studies suggesting it may obscure true tumor thickness or delay diagnosis [62]. Additionally, the sentinel lymph node status serves as an independent and robust predictor of both recurrence risk and overall survival, playing a central role in staging and management decisions [63]. In summary, although histologic subtype is not formally incorporated into current staging systems like the AJCC, it remains a vital contextual factor [64]. When considered alongside established prognostic markers, it can meaningfully inform surveillance strategies and decisions regarding adjuvant therapy [65].

Molecular and genetic alterations in CM subtypes

CM is a genetically heterogeneous malignancy, with a molecular landscape that varies substantially across histopathological subtypes [14]. Recognizing these genetic alterations is critical for prognosis, therapeutic targeting, and clinical trial selection [66]. Table 3 outlines the most common molecular alterations stratified by melanoma subtype.

**Table 3:** Molecular and genetic alterations in the melanoma subtypes SSM: Superficial spreading melanoma; NM: Nodular melanoma; LMM: Lentigo maligna melanoma; ALM: Acral lentiginous melanoma; DM: Desmoplastic melanoma; MAPK: Mitogen-activated protein kinase; TMB: Tumor mutational burden; BRAF: B-Raf proto-oncogene, serine/threonine kinase; NRAS: Neuroblastoma RAS viral oncogene homolog; KIT: KIT Proto-oncogene receptor tyrosine kinase; NF1: Neurofibromin 1.

Subtype	Common genetic mutations	MAPK pathway activation	Targetable mutations	Notes
SSM [67]	BRAF (esp. V600E), NRAS	Yes	BRAF, MEK	Responsive to BRAF/MEK inhibitors
NM [68]	NRAS, BRAF	Yes	BRAF (sometimes)	Often thicker, aggressive behavior
LMM [69]	NRAS, KIT	Yes	KIT (variable)	Less BRAF; photodamage-related mutations
ALM [70]	KIT, Chromosomal instability	Variable	KIT (sometimes)	Typically not responsive to BRAF inhibitors
DM [71]	NF1, High TMB	Yes	Rarely	Responds better to immunotherapy; S100/SOX10 positive

BRAF mutations, particularly the V600E variant, are present in approximately 40-60% of melanomas and are most frequently observed in SSM, especially those arising on intermittently sun-exposed skin [72]. These mutations activate the mitogen-activated protein kinase (MAPK) signaling pathway and are typically associated with younger patients and superficial lesions [73]. The development of BRAF and mitogen-activated protein kinase kinase (MEK) inhibitors, such as vemurafenib and dabrafenib, has significantly improved outcomes in patients with advanced BRAF-mutant disease [74]. NRAS mutations, seen in about 15-20% of cases, occur more commonly in NM and less frequently in ALM and LMM [75]. Like BRAF mutations, NRAS mutations also activate the MAPK pathway, but they are currently not directly targetable, and NRAS-mutant tumors often demonstrate greater thickness, increased mitotic activity, and more aggressive clinical behavior [75]. KIT mutations and amplifications are identified in approximately 15-20% of ALM and mucosal melanomas and in a subset of LMM [76]. These tumors may exhibit sensitivity to tyrosine kinase inhibitors such as imatinib, although clinical resistance is common [77]. In contrast, KIT mutations are rare in SSM and NM [78]. NF1 mutations are a defining feature of DM and are also seen in other melanomas associated with chronic sun exposure [79]. These mutations lead to MAPK pathway hyperactivation and are strongly correlated with a high tumor mutational burden (TMB), which may enhance responsiveness to immune checkpoint inhibitors, though targeted therapies remain limited for NF1-mutant disease [79]. Telomerase reverse transcriptase (TERT) promoter mutations, found across all melanoma subtypes, promote telomerase activity and cellular proliferation [80]. Their presence, especially when co-occurring with BRAF or NRAS mutations, is linked to more aggressive disease and worse prognosis [81]. Other notable genetic alterations include guanine nucleotide-binding protein subunit alpha Q (GNAQ) and guanine nucleotide-binding protein subunit alpha 11 (GNA11) mutations, which are more typical of uveal melanoma rather than cutaneous forms; cyclin dependent kinase inhibitor 2A (CDKN2A) deletions, commonly seen in familial melanoma and some sporadic cases, leading to cell cycle dysregulation; and phosphatase and tensin homolog (PTEN) loss, often coexisting with BRAF mutations, which can contribute to resistance against targeted therapies [82-84]. The Cancer Genome Atlas (TCGA) proposes a genomic classification of CM into four primary categories: BRAF-mutant, NRAS-mutant, NF1-mutant, and triple wild-type [85]. This classification aids in stratifying patients for clinical trials and tailoring therapeutic strategies.

Diagnostic and immunohistochemical (IHC) markers

Accurate diagnosis of CM depends primarily on meticulous histopathologic evaluation, complemented by IHC staining and, increasingly, molecular diagnostic techniques [86]. These ancillary tools are particularly valuable in diagnostically challenging cases such as amelanotic, desmoplastic, or nevoid melanomas, which can closely resemble benign or non-melanocytic lesions [87]. Among the most commonly used IHC markers, S100 stands out for its high sensitivity across virtually all melanoma subtypes, including desmoplastic variants, although it lacks specificity and thus serves best as a general screening tool [88]. SOX10, a nuclear transcription factor, offers both high sensitivity and specificity for melanocytic differentiation and is especially helpful in desmoplastic and spindle-cell melanomas [89]. Melan-A (MART-1) and HMB-45 are more specific markers but less sensitive in poorly differentiated or desmoplastic tumors; they are often used in conjunction with broader markers to confirm melanocytic origin [89]. MITF, another nuclear melanocytic marker, may aid in diagnosing ambiguous lesions [90]. The emerging marker Preferentially Expressed Antigen in Melanoma (PRAME) is gaining traction for its ability to distinguish melanomas from benign nevi, especially when used alongside conventional markers [91]. Additionally, Ki-67, a proliferation index marker, can provide insight into mitotic activity in borderline or uncertain lesions [92]. IHC interpretation must often be tailored to specific subtypes [93]. For example, DM frequently lacks Melan-A and HMB-45 expression but is reliably stained with S100 and SOX10; neurotropic variants may also express neural markers such as neurofilament [93,94]. Spitzoid melanomas often necessitate a panel of melanocytic and proliferation markers, with molecular testing (e.g., for Harvey rat sarcoma viral oncogene homolog (HRAS) mutations or anaplastic lymphoma kinase (ALK) fusions) playing a confirmatory role [95]. Nevoid and amelanotic melanomas, due to their lack of pigmentation and resemblance to benign nevi, require a careful combination of multiple IHC markers and, in some cases, molecular diagnostics for accurate classification [96]. When IHC is inconclusive, molecular diagnostic techniques can be critical [97]. Fluorescence in situ hybridization (FISH) and comparative genomic hybridization (CGH) help detect chromosomal aberrations, while next-generation sequencing (NGS) panels can identify actionable mutations in genes such as BRAF, NRAS, KIT, and NF1 [98]. Gene expression profiling is also being explored to distinguish between benign and malignant melanocytic lesions, particularly in diagnostically ambiguous settings [99]. Emerging technologies continue to enhance diagnostic capabilities [100]. Digital pathology and AI-assisted image analysis are increasingly adopted to improve the consistency and accuracy of histological interpretation [101]. Meanwhile, liquid biopsy approaches, including circulating tumor DNA (ctDNA) testing, are under investigation for their potential in early melanoma detection, disease monitoring, and recurrence prediction [101].

Clinical management and treatment strategies

The management of CM is largely dictated by tumor stage, molecular alterations, and histopathological features, with important subtype-specific considerations that influence clinical decision-making [16]. Advances in targeted therapies and immunotherapy have significantly improved outcomes, particularly in patients with advanced-stage disease [102]. Surgical excision with histologically clear margins remains the mainstay of treatment for localized melanoma [102]. The extent of wide local excision is determined by Breslow thickness [103]. For instance, 1 cm margins for tumors ≤1 mm thick, and 2 cm for those >2 mm [104]. Sentinel lymph node biopsy (SLNB) is indicated for tumors classified as ≥T1b (i.e., ≥0.8 mm with ulceration or ≥1.0 mm without ulceration), as it provides critical information for staging and prognosis [105].

Certain subtypes, such as DM, exhibit a lower rate of lymphatic spread but a higher risk of local recurrence, which may prompt consideration of adjuvant radiotherapy, particularly in neurotropic or mixed variants [13]. For patients with resected stage III melanoma, and selected cases of stage IIB/IIC, adjuvant therapy is recommended [106]. Anti-Programmed Cell Death Protein 1 (PD-1) immunotherapies such as nivolumab and pembrolizumab are standard options that have demonstrated improved recurrence-free survival [107]. In patients with BRAF V600-mutant melanoma, the combination of dabrafenib and trametinib offers a targeted adjuvant approach [108].

The decision between immunotherapy and targeted therapy depends on factors including the patient's mutation profile, age, comorbidities, and treatment preferences [109]. In the setting of stage IV or unresectable stage III disease, systemic therapy becomes central [110]. Immune checkpoint inhibitors, either anti-PD-1 alone or combined with anti-Cytotoxic T-Lymphocyte-Associated Protein 4 (CTLA-4) agents, can produce durable responses irrespective of mutational status [111]. For patients with BRAF-mutant melanoma, BRAF/MEK inhibitors induce rapid responses, although they tend to be less durable than immunotherapy [111]. Tumors harboring KIT mutations, commonly seen in acral lentiginous and mucosal melanomas, may benefit from tyrosine kinase inhibitors like imatinib, although treatment resistance is frequently observed [112]. Specific subtypes introduce unique therapeutic considerations [113]. For instance, ALM and DM often lack common driver mutations and typically respond more favorably to immunotherapy than targeted agents [114]. LMM, though typically slow-growing, can exhibit deep invasion over time, underscoring the importance of early diagnosis and intervention [114]. Spitzoid melanoma and other rare variants may necessitate individualized treatment plans based on molecular and histologic data [115].

Emerging biomarkers and future directions

As melanoma treatment continues to evolve, increasing focus has been placed on identifying biomarkers that enhance prognostication, guide therapeutic decisions, and facilitate disease monitoring [116]. While traditional histopathological parameters remain foundational, emerging molecular, genetic, and immunologic markers are reshaping the landscape of personalized melanoma care [8]. Among prognostic and predictive biomarkers, TMB has gained attention; high TMB is associated with increased neoantigen expression and may predict favorable responses to immune checkpoint inhibitors, particularly in tumors such as DM and those with chronic sun exposure [117]. Programmed Death-Ligand 1 (PD-L1) expression, although not routinely used in clinical decision-making for melanoma, can offer insight into potential responsiveness to immunotherapy, though its predictive value is limited by variability in expression and sampling challenges [118]. Gene expression profiling (GEP), using multigene panels like DecisionDx-Melanoma, is being developed to stratify patients by recurrence risk, which may help tailor surveillance intensity and adjuvant treatment decisions [119]. Liquid biopsy approaches, particularly the assessment of ctDNA, are under investigation for their ability to detect residual disease, monitor treatment response, and predict recurrence [120]. Similarly, circulating tumor cells (CTCs) and microRNAs (miRNAs) are promising tools for non-invasive disease monitoring, though their clinical application is still under validation [121].

Epigenetic and transcriptomic markers, including DNA methylation patterns, histone modifications, and profiles of non-coding RNAs such as miRNAs and long non-coding RNAs (lncRNAs), offer additional layers of tumor regulation and may play a role in disease progression and therapy resistance [121]. These changes are particularly important in tumors that lack actionable mutations, and they may eventually serve as therapeutic targets themselves [122]. The tumor microenvironment (TME) also plays a critical role in determining treatment response [123]. The density and activity of T-cell infiltration, macrophage polarization, and immune checkpoint expression influence the efficacy of immunotherapy [124]. Tumors with an “inflamed” or immunologically active TME are more likely to respond to checkpoint inhibitors, while those with a “cold” TME may require combination strategies to stimulate immune activation [125].

Finally, advances in technological platforms are contributing to more nuanced insights into tumor biology [126]. Single-cell sequencing, spatial transcriptomics, and artificial intelligence (AI) applied to digital pathology help to unravel intratumoral heterogeneity and complex microenvironmental interactions [127]. Ongoing clinical trials are exploring novel combinations of immunotherapy with epigenetic drugs and cancer vaccines, customized to individual tumor subtypes and molecular profiles [128].

Challenges and limitations in histopathological classification

Despite the long-standing utility of histopathological subtyping in CM, several limitations reduce its precision and clinical applicability [129]. Chief among these are diagnostic ambiguity, overlapping morphological features among subtypes, and limited prognostic accuracy when histology is used in isolation [130]. One of the most persistent challenges is interobserver variability; the interpretation of melanocytic lesions can be highly subjective, especially when distinguishing between early invasive melanoma and atypical nevi, desmoplastic and spindle-cell melanoma, or Spitzoid melanoma and Spitz nevus [131]. Studies have demonstrated substantial variability even among experienced dermatopathologists, underscoring the need for standardized diagnostic criteria and consensus-driven guidelines [132].

Another significant challenge is the overlap between subtypes [133]. Some melanomas exhibit mixed histologic patterns [87]. For instance, features of both SSM and NM within the same lesion [134]. Similarly, lentiginous growth may be observed in both ALM and LMM, and DM may coexist with conventional melanoma components, resulting in so-called "mixed" versus "pure" variants [135]. These overlaps can obscure prognostic and therapeutic distinctions, particularly when the subtype informs surgical planning or decisions regarding adjuvant radiation [136]. Moreover, histological classification does not always align with molecular findings [9]. For example, BRAF mutations, typically associated with SSM, may also occur in nodular or, more rarely, acral melanomas [137]. Conversely, KIT mutations, which are characteristic of ALM and mucosal melanomas, are not present in all tumors arising at acral sites [138]. Thus, relying solely on morphology risks missing actionable mutations, reinforcing the need to supplement histopathologic evaluation with molecular testing [139]. Importantly, histopathological subtype is not currently incorporated into formal staging systems like the AJCC system, which prioritizes parameters such as tumor depth, ulceration, lymph node status, and distant metastasis [64]. As a result, subtype-specific prognostic information may be underutilized in routine treatment planning, despite its clinical relevance [140]. Challenges also arise with rare melanoma subtypes, such as nevoid, Spitzoid, and primary dermal melanoma [141]. These variants are underrepresented in clinical trials and the literature, making their diagnosis and management more reliant on expert opinion or extrapolation from more common melanoma types [114]. Addressing these challenges requires a multimodal diagnostic approach that integrates histology, immunohistochemistry, molecular profiling, and clinical context [142]. Emerging technologies such as AI and digital image analysis offer promising avenues for reducing diagnostic subjectivity and enhancing reproducibility [143].

## Conclusions

Cutaneous melanoma is a biologically complex malignancy with distinct histopathological subtypes that influence presentation, prognosis, and clinical behavior. Subtypes such as SSM, NM, LMM, ALM, and DM provide valuable diagnostic and prognostic context, but they must be interpreted alongside molecular and immunologic data for optimal patient care. Advances in molecular diagnostics have enabled more personalized treatment strategies, while IHC and gene expression profiling continue to refine diagnostic accuracy, especially in rare or ambiguous cases. Emerging tools like ctDNA, epigenetic profiling, and TME analysis show growing potential for non-invasive monitoring and novel therapeutic approaches. Despite the utility of the current classification systems, challenges remain, including interobserver variability, overlapping features, and the limited characterization of rare subtypes. These limitations highlight the need for a multimodal approach that integrates histopathology, molecular testing, and clinical context. In the era of targeted and immune-based therapies, understanding the broader prognostic and biological implications of melanoma subtypes is essential, not only for academic purposes but as a critical step toward improving survival and quality of life for patients facing this aggressive disease.
